# Clinicopathological characteristics and clinical outcomes of remnant gastric cancer with endoscopic submucosal dissection

**DOI:** 10.3389/fonc.2025.1639531

**Published:** 2025-09-22

**Authors:** Xiang-Yu Chen, Jing Wang, Zi-Yu Li, Qi Wu

**Affiliations:** ^1^ Key Laboratory of Carcinogenesis and Translational Research (Ministry of Education), Endoscopy Center, Peking University Cancer Hospital & Institute, Beijing, China; ^2^ Key Laboratory of Carcinogenesis and Translational Research (Ministry of Education), Gastrointestinal Cancer Center, Peking University Cancer Hospital & Institute, Beijing, China; ^3^ State Key Laboratory of Holistic Integrative Management of Gastrointestinal Cancers, Endoscopy Center, Peking University Cancer Hospital & Institute, Beijing, China

**Keywords:** endoscopic submucosal dissection, gastrectomy, gastric stump, remnant gastric cancer, *en bloc* resection

## Abstract

**Background:**

Remnant gastric cancer is distinct from primary gastric cancer clinically and pathologically. Recently, endoscopic submucosal dissection, widely used for treating early gastric cancer, has also been used to treat remnant gastric cancer. However, owing to the previous surgeries, endoscopic resection of remnant gastric cancer is more complicated. This study aimed to elucidate the clinicopathological characteristics of remnant gastric cancer and evaluate clinical outcomes of endoscopic submucosal dissection for this condition.

**Methods:**

This retrospective study examined the clinicopathological characteristics and clinical outcomes in 30 patients (32 lesions) with remnant gastric cancer who underwent endoscopic submucosal dissection from 2012 to 2023 at the Endoscopy Center of Peking University Cancer Hospital, China.

**Results:**

The primary disease was a malignant tumor in 25 patients and a benign tumor in 4. Billroth II was the major reconstruction method used in the initial surgery. The median interval from previous surgery to remnant gastric cancer detection was 4.2 years. The mean endoscopic submucosal dissection time was 136 ± 71 min. The *en bloc*, R0, and curative resection rates were 96.9%, 78.1%, and 71.9%, respectively. While one patient had a perforation during the procedure, none experienced delayed postoperative bleeding. Two patients had local recurrence, and five died during the follow-up. The 5-year overall survival rate was 83.0%.

**Conclusions:**

Remnant gastric cancer development is influenced by the type of initial disease and prior surgery. Endoscopic submucosal dissection is a safe and effective treatment for early remnant gastric cancer, with potential applicability for certain non-early-stage lesions. However, being technically challenging, endoscopic submucosal dissection requires the skills of experienced endoscopists and careful evaluation.

## 1 Introduction

Gastric cancer (GC) is the fifth most common cancer worldwide, with the highest incidence in East Asia (1). Remnant gastric cancer (RGC) is a type of GC with specific clinical features and prognoses, accounting for 1–2% of all GC cases (2). The concept of RGC was first proposed by Balfour in the early 20th century to describe malignant tumors arising in the remnant stomach after surgery for benign ulcers (3). However, with the widespread use of anti-acid medications in recent decades, the number of patients undergoing surgery for ulcers has significantly decreased (2, 4), although the development of RGC after surgery for malignant tumors has become more common. Owing to the long interval between benign ulcers and RGC, as well as the improved prognosis of GC, RGC incidence has increased in recent years (5). RGC is often diagnosed at an advanced stage, leading to a poor prognosis (6). No standard treatment exists for RGC, and surgery is typically the first-line therapy in clinical practice. Although the efficacy of surgical treatment has been recognized, the long-term prognosis in patients with RGC after surgery remains unsatisfactory. The curative resection rate for RGC is 69–70.7%, with a 5-year survival rate of 25–56% and complication rate of 19–46.6% (7–11). Initial surgery often leads to adhesions between the remnant stomach and surrounding tissues or organs, making a second surgery challenging with a higher risk of postoperative complications (5).

In recent years, some centers have performed endoscopic resections of RGC. Endoscopic submucosal dissection (ESD) allows *en bloc* resection of lesions with large diameters and irregular shapes, leading to better clinical outcomes for RGC compared to endoscopic mucosal resection (12). Previous studies have reported ESD for RGC achieving favorable *en bloc* (90–100%), R0 (77–94%), and curative (71–84.6%) resection rates (13–17). However, factors such as mucosal fibrosis, the presence of surgical staples, limited space, and loss of pyloric function make ESD for RGC more technically challenging (12, 18). Although endoscopic resection has become the standard treatment for early GC in many countries (19, 20), guidelines and high-grade evidence for its application in RGC are lacking. Due to the characteristics of the RGC development, the number of patients with RGC included in previous studies was generally limited. Furthermore, some clinical features and the pathogenesis of RGC remain unclear owing to its relative rarity. For example, it is unclear what clinicopathological factors affect RGC initiation. Therefore, in this study, we aimed to evaluate the safety, feasibility, and effectiveness of ESD for RGC. To this end, we analyzed the clinicopathological characteristics and outcomes in patients with RGC who underwent ESD at our endoscopy center over the past 11 years.

## 2 Materials and methods

### 2.1 Study design and patients

This retrospective study included patients with RGC who underwent ESD at Peking University Cancer Hospital in China from 2012 to 2023. Based on the Japan Gastric Cancer Association (JGCA) definition (21), RGC included all carcinomas in the remnant stomach regardless of the initial disease, reconstruction methods, and time interval, including primary RGC after benign disease, synchronous multiple gastric cancer (SRGC), metachronous multiple gastric cancer (MRGC), and local recurrence. MRGC was defined as cancer detected 12 months or more after the primary surgery, developed independently from the primary cancer, to distinguish it from SRGC (new neoplasm found within 12 months after surgery) and local recurrence (cancer associated with prior surgery, <1 cm from the initial lesion). We collected the clinicopathological characteristics, *en bloc* resection rates, R0 resection rates, curative resection rates, operative times, postoperative complications (perforation and delayed bleeding), recurrence, and survival information. All patients underwent a preoperative endoscopic examination and pathological biopsy. Patients with (1) benign disease or low intraepithelial neoplasia in postoperative pathology and (2) incomplete medical records were excluded from the study. Each patient underwent preoperative magnifying endoscopy to assess the lesion range, differentiation degree, and infiltration depth, along with enhanced CT to exclude lymph node metastasis or distant metastasis. Endoscopic ultrasonography (EUS) was used to diagnose suspected submucosal invasive lesions. The indications for ESD in patients with RGC were based on the JGCA guidelines and classified as absolute, relative, or outside indications (22).

Absolute indication: (1) differentiated-type intramucosal cancer (T1a) without ulcerative findings (UL0); (2) differentiated-type intramucosal cancer (T1a) with ulcerative findings (UL1), and the diameter is ≤3 cm; and (3) undifferentiated-type intramucosal cancer (T1a) without ulcerative findings (UL0), and the diameter is ≤2 cm.

Older and high-operative-risk patients with multiple comorbidities were considered suitable for relative indications. Tumors with outside indications did not fulfill the criteria for absolute or relative indications.

This study was approved by the Ethics Committee of Peking University Cancer Hospital (2015KT44). Written informed consent was obtained from all patients.

### 2.2 ESD procedure

**Figure 1:**
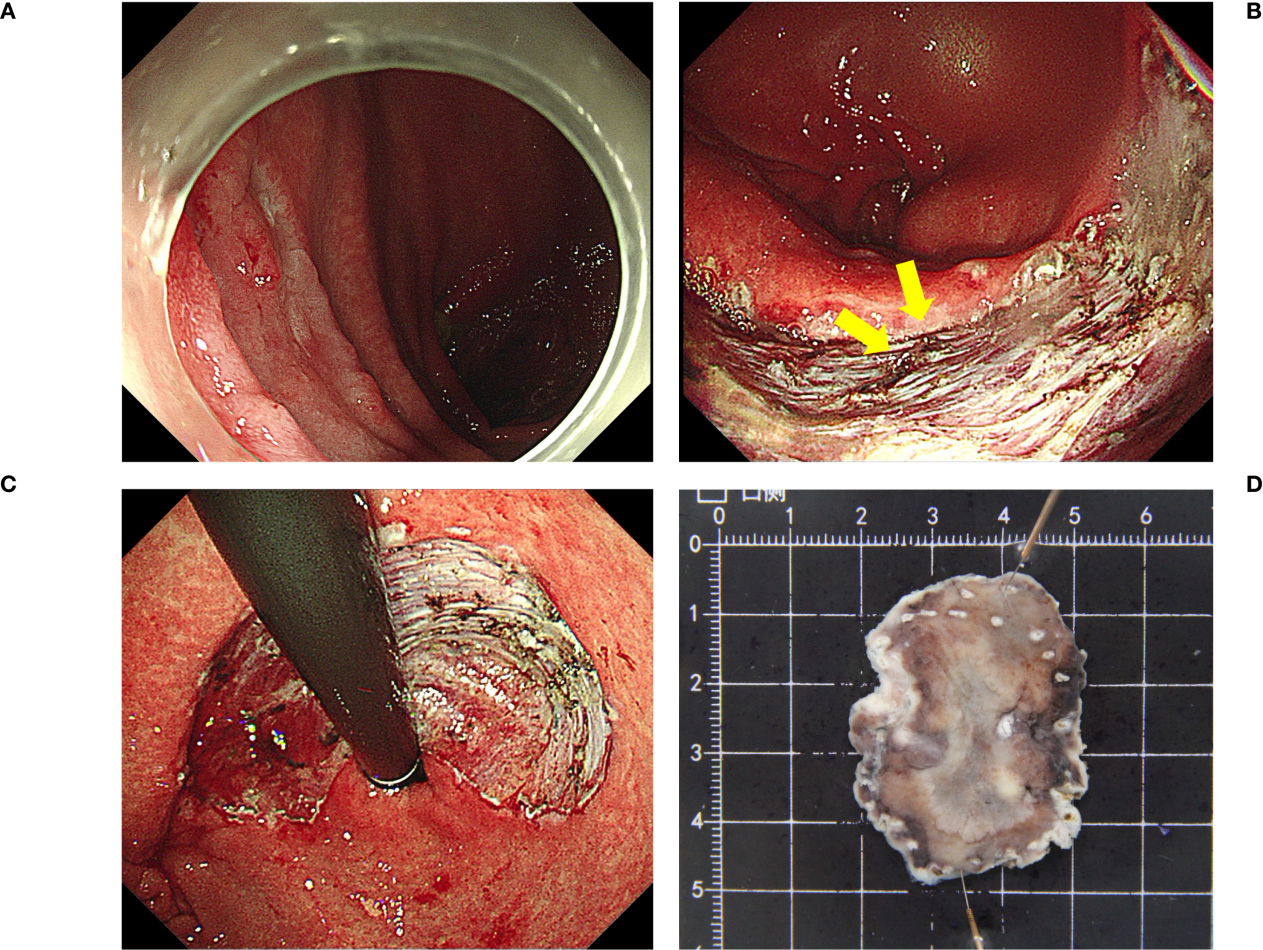
The ESD procedure **(A)** A remnant gastric tumor confirmed at the posterior part of the stomach body; **(B)** Surgical staples (yellow arrows). **(C, D)** *En bloc* resection. Pathological results: tub1>tub2, type 0-IIa, 3.3×2.3 cm, M, ly0, V0, UL0, HM0, VM0. ESD, endoscopic submucosal dissection.

ESD was performed by an experienced endoscopist who had performed more than 100 ESDs per year for the last 5 years. ESD was performed using a single-channel upper gastrointestinal endoscope (GIF Q260J; Olympus Corporation) and an electrosurgical unit (VIO 200S; ERBE Elektromedizin GmbH, Tübingen, Germany) (**Figure 1**). A premixed sterilized solution of glycerol (10% glycerol and 5% fructose, Cisen Pharmaceutical, Co., Ltd.) with indigo carmine and epinephrine was injected submucosally using an injection needle (NM-200L-0423; Olympus Corporation). After marking the lesion margin, a mucosal incision and submucosal dissection were performed using a Dual Knife (KD-650U; Olympus Corporation). During dissection, extensive fibrosis and surgical staples were found in the suture line. For the staple that was difficult to avoid, we used the tip of a pointed tip-type knife such as Dual Knife to stick it on the staple, and switched to the cutting mode to remove the staple. Regarding the dissection of fibrotic mucosa, we first incised the mucosa in the peripheral area where fibrosis had not yet formed. Then, we performed deep dissection and separation of the submucosa, using this dissection depth as a reference for handling the fibrotic mucosa. During this process, we used the Dual Knife to increase the cutting efficiency and precision of the fibrotic mucosa, avoiding cutting into the muscularis mucosa or the muscularis propria.

### 2.3 Histological evaluation

After fixation in 10% formalin, the resected specimens were evaluated pathologically in 2-mm-thick sections. Tumor size, invasion depth, ulceration or ulcer scarring, horizontal and vertical tumor margins, and the presence of lymphatic and vascular infiltration were assessed to determine the curability of ESD. En bloc resection involved removing the entire lesion in a single piece. En bloc resection with negative horizontal and vertical margins was defined as R0 or complete resection. Curative resection was performed according to the Japanese Gastric Cancer Treatment Guidelines (22), including eCura A and eCura B, as shown in **Table 1**.

**Table 1:** JGCA evaluation of curability.

HM0, VM0, ly0, V0, en bloc resection, and any of the following conditions are fulfilled:	
eCuraA	UL0, any tumor size, differentiated, pT1a
	UL1, tumor size ≤3 cm, differentiated, pT1a
	UL0, tumor size ≤2 cm, undifferentiated, pT1a
eCuraB	Tumor size ≤3 cm, differentiated, pT1b1(SM1^a^)
eCuraC	Fulfill neither eCuraA nor eCuraB

JGCA, Japan Gastric Cancer Association; UL0, no ulcerative findings; UL1, ulcerative findings; HM0, negative horizontal margin; VM0, negative vertical margin; Ly0 V0, no lymphovascular infiltration.

a less than 500 μm from the muscularis mucosa.

### 2.4 Complications and recurrence

Postoperative delayed bleeding was identified based on obvious clinical symptoms such as hematemesis, melena, and hemoglobin decrease >20 g/L or endoscopically visible bleeding requiring hemostatic measures. Perforation was defined as a muscle defect requiring a metal clip suture or the presence of free air beneath the diaphragm on postoperative radiography or computed tomography. Local recurrence was defined as the occurrence of tumor lesions at the primary resection site or within 1cm more than 6 months postoperatively.

### 2.5 Statistical analysis

Continuous variables are expressed as the mean ± standard deviation or median (interquartile range [IQR]). Cumulative survival rates were calculated using the Kaplan–Meier curve. *P* value of <0.05 was considered to be significant. The kappa coefficient was used in the agreement analysis. A kappa value of 1 indicates perfect agreement, and a value of 0 indicates no agreement beyond chance. Kappa values of 0.4–0.5, 0.5–0.6, and >0.6 indicates moderate, good, and high levels of agreement, respectively. All data were analyzed using SPSS (version 25.0) (Armonk, NY).

## 3 Results

### 3.1 Clinicopathological characteristics

**Table 2** summarizes the clinicopathological characteristics of the 30 patients (mean age: 65.8±7.4 years) included in the study. Most were male patients (26/30, 86.7%). The median interval from the initial surgery to the detection of remnant gastric lesions was 4.2 (IQR: 9.2–34.8) years. Previous surgeries included distal gastrectomy (n=20, 66.7%), proximal gastrectomy (n=6, 20.0%), partial gastrectomy (n=2, 6.7%), and esophagectomy with a gastric conduit (n=2, 6.7%). Distal gastrectomy with Billroth II reconstruction was the primary type of surgery performed (14/30, 46.7%). While 25 (83.3%) patients had malignant tumors as the primary disease, 4 (13.3%) had benign tumors, and 1 (3.3%) had a gastrointestinal stromal tumor.

**Table 2:** Clinicopathological characteristics of the 30 patients with RGC.

Characteristic	(n=30)
Age, years	
Mean ± SD	65.8 ± 7.4
Sex, n (%)	
Male	26 (86.7%)
Female	4 (13.3%)
Previous surgery, n (%)	
Distal gastrectomy	20 (66.7%)
Proximal gastrectomy	6 (20.0%)
Partial gastrectomy	2 (6.7%)
Esophagectomy	2 (6.7%)
Reconstruction, n (%)	
Billroth I	6 (20.0%)
Billroth II	14 (46.7%)
GEA	8 (26.7%)
Partial resection	2 (6.7%)
Primary disease, n (%)	
Benign	4 (13.3%)
Malignant	25 (83.3%)
GIST	1 (3.3%)
Interval time, year	
Median (IQR)	4.2 (9.2–34.8)

RGC, remnant gastric cancer; SD, standard deviation; GEA, gastroesophageal anastomosis; GIST, gastrointestinal stromal tumor; IQR, interquartile range.

The clinicopathological characteristics of the 32 RGC lesions are presented in **Table 3**. The mean lesion size was 20.8 ± 11.6 mm. Macroscopically, most lesions were type 0–II (27/32, 84.4%), and histologically, most were differentiated (29/32, 90.6%). While 24 (75.0%) lesions were limited to the mucosa, 8 (25.0%) showed submucosal invasion. The indications for ESD were absolute in 24 (75.0%) lesions, relative in 1 (3.1%), and external in 7 (21.9%). Thirteen (40.6%) lesions were located at the anastomosis site or linear stapling line. The agreement analysis of preoperative invasion depth assessment and postoperative pathology for 32 lesions is shown in **Table 4**. While 28 lesions (87.5%) were consistent, there were 4 inconsistent cases, of which 3 had preoperative diagnoses indicating lesions limited to the mucosa (M) and postoperative pathology confirmed involvement of the submucosa (SM), and one lesion with a preoperative diagnosis of M was found to be SM postoperatively. The Kappa coefficient was 0.636, with a *P*-value of <0.001.

**Table 3:** Clinicopathological characteristics of the 32 RGC lesions.

Characteristic	(n=32)
Macroscopic type, n (%)^a^	
0-I	5 (15.6%)
0-IIa	5 (15.6%)
0-IIb	1 (3.1%)
0-IIc	10 (31.3%)
0-IIa+IIc	11 (34.4%)
Depth, n (%)	
M	24 (75.0%)
SM	8 (25.0%)
Histological type, n (%)	
Differentiated	29 (90.6%)
Undifferentiated	3 (9.4%)
Location, n (%)	
Anastomosis or stapling line	13 (40.6%)
Remnant stomach	19 (59.4%)
Indication of ESD, n (%)	
Absolute	24 (75.0%)
Relative	1 (3.1%)
Outside	7 (21.9%)
Tumor size (mm)	
Mean ± SD	20.8 ± 11.6

RGC, remnant gastric cancer; SM, submucosa; M, mucosa.

a as per the Japanese Classification of Gastric Carcinomas.

**Table 4:** Agreement analysis of preoperative invasion depth assessment and postoperative pathology for 32 RGC lesions.

		Postoperative pathology		Total
		M	SM	
Preoperative assessment	M	23	3	26
	SM	1	5	6
Total		24	8	32

SM, submucosa; M, mucosa.

Kappa Value=0.636, P<0.001.

### 3.2 Feasibility and safety analysis of ESD for RGC

The mean operation time for ESD was 136 ± 71 min. The *en bloc* resection rate was 96.9%, and only one lesion, which was present in the submucosa, was unresectable. The R0 and curative resection rates were 78.1% and 71.9%, respectively. The perforation rate was 3.1%, with one case of perforation during the operation. There were no cases of delayed bleeding post-resection. eCuraA was achieved in 22 (68.8%) lesions, eCuraB in 1 (3.1%), and eCuraC in 9 (28.1%). **Table 5** summarizes the outcomes and complications of ESD for RGC.

**Table 5:** Treatment outcomes and complications of ESD for RGC.

Outcome	N=32
Operative time (min)	
Mean ± SD	136 ± 71
En bloc resection, n (%)	31 (96.9%)
R0 resection, n (%)	25 (78.1%)
Curative resection, n (%)	23 (71.9%)
Perforation, n (%)	1 (3.1%)
Delayed bleeding, n (%)	0 (0.0%)
eCuraA, n (%)	22 (68.8%)
eCuraB, n (%)	1 (3.1%)
eCuraC, n (%)	9 (28.1%)

ESD, endoscopic submucosal dissection; RGC, remnant gastric cancer.

### 3.3 Recurrence and survival analysis

**Figure 2:**
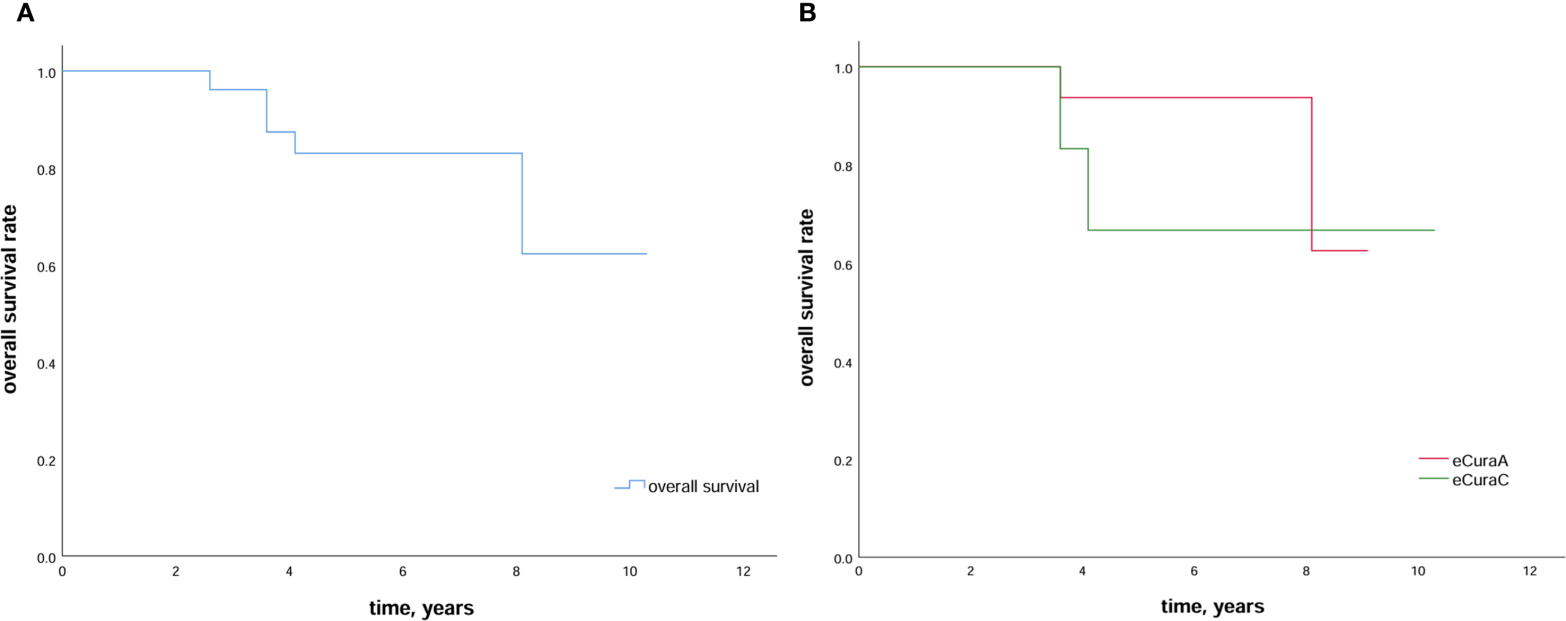
Survival outcomes after ESD **(A)** Kaplan–Meier estimation of survival in patients who undergo ESD in a remnant stomach or a gastric conduit. The 5-year overall survival is 83.0%; **(B)** Kaplan–Meier curve comparing the 5-year overall survival rate in patients with RGC subjected to eCuraA and eCuraC resection. ESD, endoscopic submucosal dissection; RGC, remnant gastric cancer.

During the follow-up period, two of 30 patients experienced local recurrence in months 10 and 22 after ESD. While one of these patients underwent radical gastrectomy and survived without recurrence after surgery, the other received adjuvant therapy (chemotherapy and targeted therapy) but died in month 49 following ESD. By the end of the follow-up period, five patients had died: two from GC, two from other diseases, and one from an unknown cause. The 5-year overall survival rate was 83.0%, and 5-year overall survival rates in patients with eCuraA and eCuraC tumors were 93.8% and 66.7%, respectively (**Figure 2**). There was only one eCuraB lesion, so we did not perform survival curve analysis for this patient group.

Detailed information on the five deaths is presented in **Table 6**. Of the two patients who died from GC, one had a relative indication for ESD, and liver metastases were discovered during the initial GC. After gastrectomy for the primary gastric cancer (PGC), the patient underwent radiofrequency ablation and transarterial chemoembolization (TACE) several times for the liver lesions. The RGC lesion developed during TACE, and the patient was treated when the liver metastatic lesions had stabilized. However, the postoperative horizontal and vertical margins were both pathologically positive, and local recurrence was seen 10 months post-ESD. The patient died of multiple metastases from GC 49 months post-ESD. Another patient who died from GC had absolute indications, achieved curative resection with ESD, and died of lung metastases from GC in postoperative month 31.

**Table 6:** Details of the five deaths in the study.

Case	1	2	3	4	5
Sex	Male	Male	Male	Male	Male
Age	75	64	61	81	68
Time to death, month	97	43	43	49	31
Reason of death	Liver cirrhosis	Pancreatic cancer	Unknown	Recurrence (multiple)	Recurrence (lung)
Ulcer	(-)	(+)	(-)	(-)	(-)
Lymphovascular infiltration	Ly0, V0	Ly0, V0	Ly0, V0	Ly0, V0	Ly0, V0
Margin	HM0, VM0	HM0, VM1	HM0, VM0	HM1, VM1	HM0, VM0
Curability	eCuraA	eCuraC	eCuraA	eCuraC	eCuraB
Indication	Absolute	Outside	Absolute	Relative	Outside
Location	NA	NA	NA	A	NA
Primary disease	GC	GC	Ulcer	GC	GC
Histology	D	U	D	D	D
Depth	M	SM	M	SM	SM
Macroscopic type	0-IIa	0-IIc	0-IIa+IIc	0-I	0-IIc

D, differentiated; U, undifferentiated; GC, gastric cancer; NA, non-anastomosis; A, anastomosis.

## 4 Discussion

This study analyzed the clinicopathological characteristics of patients with RGC and evaluated the outcomes, complications, and long-term prognosis of ESD in these patients. In most patients, the primary cause of RGC was malignant tumors, mainly GC. During the initial surgery, distal gastrectomy with Billroth II reconstruction was the primary surgical method used. The median interval between the primary surgery and detection of remnant gastric lesions was 4.2 (IQR: 9.2–34.8) years. ESD for RGC achieved high *en bloc*, R0, and curative resection rates while maintaining a low complication rate and favorable long-term prognosis. Our findings, therefore, suggest that ESD is a safe, feasible, and effective treatment for RGC.

The factors influencing the development of RGC after surgery for benign and malignant tumors vary. Long-term stimulation by duodenal reflux has been reported to be one of the major causes of RGC after benign diseases (RGCB), resulting in mucosal inflammation and regeneration (4, 6, 23). In contrast, RGC after malignant tumors (RGCM) mainly arises from the progression of preexisting mucosal changes (such as atrophic gastritis, intestinal metaplasia, and dysplasia). The shorter interval between the initial surgery and RGCM compared with RGCB supports this perspective. Furthermore, the reconstruction method used during initial surgery influences the development of RGC (2). Compared with Billroth I reconstruction, Billroth II reconstruction is more frequently associated with postoperative duodenal reflux, leading to RGC arising from the anastomosis sites. In this study, the median interval between initial surgery and RGCM was 4.0 (IQR: 6.4–14.6) years, while the mean interval for RGCB development was 31.5 ± 14.7 years. Thirteen patients underwent Billroth II reconstruction, and of them, nine (69.2%) had RGC lesions at the anastomosis or linear stapling line, higher than Billroth I reconstruction (3/6, 50.0%), consistent with the previous findings.

The lymphatic drainage pathways of RGC differ from those of PGC and vary depending on the initial disease type (24, 25). The lymph node metastasis (LNM) rate in RGC after the benign disease is higher than that in RGCM tumors because patients with the latter undergo lymph node dissection during initial surgery (26–28). Endoscopic resection is feasible for PGC lesions limited to the mucosa due to their low LNM rates (19). For RGCs that have undergone lymph node dissection during initial surgery, the LNM rate may be lower than that for PGC (29). Therefore, the indication criteria for ESD in RGC should be expanded. Compared with other studies, we included a higher proportion of patients with outside indications (7/30, 21.9%); of them, six (85.7%), three (42.9%), and one (14.3%) underwent *en bloc*, R0, and curative resections, respectively. Although statistical comparison with other indication groups was not possible due to the limited sample size, our findings suggest that patients with the outside indications could achieve favorable *en bloc* and R0 resections under certain conditions. Nishide et al. compared the clinical outcomes of ESD for RGC with different indications (standard, expanded, and outside of criteria) and found no significant differences in *en bloc* resection, R0 resection, or complication rates (13), supporting our viewpoint. Previous studies used ESD indication criteria for RGC similar to those used for PGC (12, 13). Choi et al. indicated that RGC and PGC can use the same indication criteria for ESD based on postoperative LNM rates (30). However, research on the indication criteria for ESD for RGC is rare, and there is no consensus on whether the indications for PGC are applicable to RGC. At our center, we ruled out lymph node or distant metastasis for lesions suspected to have submucosal infiltration (cT1b) based on endoscopy and CT scans. In these cases, we opted for ESD resection after multi-disciplinary treatment (MDT) discussions and thorough communication with the patient. Although the patients with additional indications included in our study had a favourable *en bloc* resection rate, the curative resection rate was low. Among these seven patients, four had positive surgical margins postoperatively (all SM2), and by the end of follow-up, one died due to RGC recurrence. Therefore, for lesions with T1b-SM2, the indications for endoscopic resection of RGC should be expanded with caution.

RGCs located at the anastomosis or linear stapling line pose a greater challenge for ESD because of the presence of surgical staples from the initial surgery and severe local mucosal fibrosis. In our study, 13 lesions were located at the linear stapling line. *En bloc* resection was successful in all 13 (100%) lesions, while R0 resection was possible in nine (69.2%) of them, with a mean operative time of 158 ± 76 min. Compared to lesions in non-stapled sites, they had a longer operative time and lower R0 resection rate (without significant difference), which was consistent with the conclusions of previous studies (16). Surgical staples not only make ESD difficult but also exacerbate local mucosal inflammation and promote gastric carcinogenesis. Suzuki suggested that removing surgical staples during ESD for RGC at the anastomosis or suture line was safe and effective, because it could reduce specimen damage, lower postoperative complication rates, and shorten the operation time (31). Therefore, during ESD involving surgical staples, we recommend fully exposing and removing the staples during the procedure. However, removing staples extended the surgical time (158 ± 76 min vs. 136 ± 71 min) as lesion resection at the anastomosis site and staple removal are technically challenging. Endoscopists without surgical experience should carefully assess the risk of complications from ESD procedures that involve surgical staples.

Accurate evaluation of the mucosal invasion depth of early gastric cancer lesions is crucial for guiding subsequent clinical treatment. Due to postoperative mucosal fibrosis and changes in anatomical structures, estimating the invasion depth of RGC lesions becomes more challenging. In this study, the consistency rate between preoperative and postoperative diagnoses was 87.5%, with a Kappa coefficient of 0.636 (*P* < 0.001). All patients underwent magnifying endoscopy prior to ESD, and the majority (27/30, 90%) underwent EUS evaluation. EUS is critical for assessing the invasion depth of early GC (32), but its accuracy in evaluating the RGC lesions decreased due to mucosal fibrosis. This is particularly true for lesions at the anastomosis site or stapling lines, where postoperative changes in the mucosal surface and anatomical structures can lead to underestimation of the infiltration depth (33). All seven cases with additional indications in this study were due to postoperative pathology, indicating that the lesion infiltrated the submucosa. Based on EUS, two of these lesions were assessed as uT1b, two were suspected of being uT1b, and three were classified as uT1a, indicating that three cases had preoperative underestimation of infiltration depth. At the same time, one case was diagnosed as uT1b by preoperative EUS but was found to be pT1a postoperatively. According to a previous study from our cener (34), the tumor location and the presence of ulcers influence the accuracy of EUS in assessing infiltration depth. Lesions located in the lower stomach tend to get overestimated (uT1b might eventually be reclassified as pT1a). For an early GC located in the lower stomach without ulceration, further evaluation or diagnostic ESD is recommended. However, research on the factors affecting the accuracy of preoperative EUS evaluation for RGC lesions is still lacking. Therefore, for lesions crossing the anastomosis, where the submucosa was adhered, we used magnifying endoscopy to assess the infiltration depth. If it suggested a potential invasion of the muscularis propria, we confirmed it using EUS. Combining multiple endoscopic evaluation techniques helps in the accurate preoperative assessment of RGC.

Different reconstruction methods result in anatomical and physiological changes that directly impact the technical feasibility and risk stratification of ESD for RGC. For example, after PG, ESD becomes challenging due to the mucosal fibrosis caused by reflux esophagitis and the narrowing of the anastomosis. Lesions located at the pseudo-fornix of the remnant stomach were often not suitable for endoscopic resection (35). After DG, reflux of duodenal contents (bile and pancreatic fluid) leads to remnant gastric inflammation and submucosal neovascularisation, making ESD dissection prone to bleeding (36). Meanwhile, after Billroth II reconstruction, remnant gastric lesions were more likely to occur at the anastomotic suture line, leading to lower R0 resection and curative resection rates after DG than PG (37). ESD was the most difficult after esophagectomy due to the restricted working space caused by the elongated gastric tube, unusual fluid pooling areas, and multiple suture lines and staples, increasing the risk of perforation during the procedure (38–40). We included two cases of RGC after esophagectomy, both of which achieved R0 resection, with an average surgical time of 42 ± 3 min, significantly shorter than PG (136 ± 71 min) and DG (173 ± 62 min). This was likely due to the small number of gastric conduit lesions (n=2), with tumor lengths all below 20 mm and located away from the anastomosis site, thus making ESD less difficult. Tumor length (median tumor size ≥ 20 mm) and the location of the lesion at the suture line were risk factors for ESD of lesions after esophagectomy (41, 42). Therefore, ESD for RGC after esophagectomy requires more careful consideration as the risk of complications such as perforation is higher. Risk stratification based on different reconstruction methods helps assess the difficulty of ESD and guide treatment decision-making.

In this study, two patients died due to GC recurrence (patients 4 and 5). Patient 4 had liver metastasis at the time of the initial surgery, and it was a palliative procedure. Due to old age and multiple comorbidities (coronary artery disease, hypertension, malnutrition, etc.), after stabilizing the liver lesions with systemic therapy, TACE, and MDT discussion, we performed ESD for the remnant gastric lesion at the anastomosis. Although this patient experienced RGC recurrence 4 months after ESD, initial GC progression and widespread metastasis were considered the primary causes of death. Patient 5 developed liver metastasis 1 year after ESD for remnant gastric lesions and ultimately died of multiorgan metastasis (liver, lung, bone). The preoperative mucosal infiltration depth of Patient 5 was T1a, but the postoperative pathology indicated T1b-SM1. Despite this, the patient still achieved a curative resection. RGC patients typically have a higher risk of metachronous recurrence and distant metastasis (43). Nonaka et al. showed that even patients with curative resection had a 7.9% metachronous gastric cancer occurrence rate within 5 years (8/101) (14). Therefore, after ESD, an individualized, long-term, and high-frequency follow-up plan should be developed to detect recurrences or new lesions early. For complex cases, such as that of patient 4, the risks and benefits of ESD should be carefully evaluated in MDT discussions.

Few studies have described adjuvant therapies for RGC that are similar to those for PGC in clinical practice. Although there is no strong evidence indicating that *Helicobacter pylori* can promote RGC development, its eradication significantly improves inflammation and pH levels in the remnant gastric cavity (2). The pathogenetic mechanisms of RGC differ from those of PGC (29), and further research is needed to explore potential treatment methods for RGC. Patients with RGC with external indications, such as those classified as T2 but who have undergone lymph node dissection during the initial surgery, have a low LNM rate. Endoscopic full-thickness resection is possible in such cases and requires an MDT discussion and careful evaluation. Recent advances in laparoscopic and endoscopic cooperative surgery have drawn attention to the treatment of GC (44, 45) and may become feasible options for RGC with outside indications in the future. In this study, a survival analysis was performed in patients with RGC who underwent different eCura resections (**Figure 2**). Patients with eCuraA demonstrated significantly higher 5-year survival rates than those in the other two groups. We could only compare the survival curves between patients with eCuraA and eCuraC tumors owing to the limited number (n=1) of those with eCuraB tumors, and no significant difference was found. Therefore, further research is required to determine whether the eCura system is suitable for evaluating the efficacy of ESD in RGC.

This study has some limitations. First, being a retrospective study, selection and information biases were unavoidable. Second, it was a single-center study with a limited sample size, and all procedures were performed by an experienced endoscopist at our center, potentially leading to selection bias and operator dependency. Third, non-cancer deaths (2/5 mortalities) and missing follow-up information (one death due to an unknown reason) may confound survival analysis. Therefore, multicenter prospective studies are needed to further evaluate the efficacy of ESD in the treatment of RGC.

In conclusion, RGC has specific clinicopathological characteristics, and the initial disease and prior surgery influence its development. ESD is a safe, feasible, and effective treatment for RGC. The study also suggested the potential applicability of ESD for removing certain non-early-stage RGC lesions. However, for lesions adjacent to surgical staples or those with deep infiltration, ESD is technically challenging and requires a comprehensive evaluation by experienced endoscopists and multi-disciplinary team. Further research is needed to investigate the pathogenic mechanisms of RGC and establish a standard treatment strategy.

## Data Availability

The original contributions presented in the study are included in the article/supplementary material. Further inquiries can be directed to the corresponding author.
